# AI-supported teaching and student engagement: the mediating role of perceived competence in vocational education

**DOI:** 10.3389/fpsyg.2026.1870826

**Published:** 2026-07-13

**Authors:** Linlin Yin, Yi Tang, Xiaoyu Ou

**Affiliations:** School of Education, Urban Vocational College of Sichuan, Chengdu, China

**Keywords:** AI-supported teaching, perceived autonomy, perceived competence, quasi-experimental design, Self-Determination Theory, student engagement, vocational education

## Abstract

**Introduction:**

Artificial intelligence (AI) is increasingly used in education, yet less is known about the psychological processes through which AI-supported teaching relates to student engagement, especially in vocational education. Drawing on Self-Determination Theory, this study examined the mediating roles of perceived competence and perceived autonomy in the relationship between AI-supported teaching and student engagement.

**Methods:**

A 10-week quasi-experimental study was conducted with 148 second-year vocational college students, assigned to either an AI-supported teaching group (*n* = 74) or a conventional instruction group (*n* = 74). Multimodal data were collected, including academic assessments, performance evaluations, self-report measures of perceived competence, perceived autonomy, and engagement, as well as behavioral records from AI-supported learning activities. Mediation analysis was performed using bootstrapping with 5,000 resamples.

**Results:**

Students in the AI-supported teaching group showed higher levels of student engagement than those in the conventional instruction group (Cohen's d = 0.84). Perceived competence showed a statistically significant indirect association between AI-supported teaching and student engagement [β = 0.22, 95% CI (0.13, 0.31)], supporting H2. In contrast, perceived autonomy did not show a statistically significant indirect effect [β = 0.02, 95% CI (−0.02, 0.06)], and therefore H3 was not supported. The direct association between AI-supported teaching and engagement remained significant after accounting for both mediators.

**Discussion:**

These findings suggest that competence-related experiences may represent an important psychological pathway linking AI-supported teaching and student engagement in structured vocational education contexts. The non-significant role of perceived autonomy indicates that motivational processes in AI-supported learning may vary across instructional settings and should be interpreted in relation to contextual and pedagogical conditions. Given the quasi-experimental design and the context-specific sample, the findings should be interpreted cautiously and further examined through larger, multi-site, andlongitudinal studies.

## 1 Introduction

The integration of artificial intelligence (AI) into educational settings has accelerated rapidly in recent years, offering new possibilities for personalized learning, real-time feedback, and data-driven instructional decision-making (Holmes et al., 2019; Kasneci et al., 2023; Zawacki-Richter et al., 2019). In vocational education, where the primary objective is to develop job-relevant practical skills, AI-supported teaching has shown particular potential. By providing adaptive scaffolding, immediate feedback, and continuous performance monitoring, AI systems can align closely with the structured and competency-based nature of vocational training (Chen et al., 2023; OECD, 2021).

Despite this growing interest, most existing research has focused on the direct effects of AI-supported teaching on learning outcomes, such as test scores and skill acquisition (e.g., Demszky et al., 2024; Ifenthaler and Schumacher, 2016). Comparatively less attention has been paid to the psychological mechanisms through which AI-supported teaching influences student engagement. This limitation is significant, as student engagement—defined as the quality of learners' active involvement in academic activities—is a critical predictor of learning effectiveness, persistence, and long-term development.

Recent studies have begun to examine the motivational and engagement-related outcomes of AI-supported learning environments. For example, Huang et al. (2026) reported that an AI-supported assessment and feedback system was associated with higher levels of learning engagement, behavioral participation, and skill performance by providing real-time feedback and adaptive support. Similarly, Hsia et al. (2025), drawing on Self-Determination Theory, found that AI-supported intelligent tutoring systems enhanced learning engagement through competence-supportive instructional mechanisms. These findings suggest that AI-supported learning environments may influence not only learning outcomes but also the motivational processes underlying student engagement. However, the psychological mechanisms through which AI-supported teaching relates to engagement remain insufficiently examined in vocational education contexts.

Self-Determination Theory (SDT; Ryan and Deci, 2000, 2020) provides a useful framework for understanding these mechanisms. SDT posits that the satisfaction of basic psychological needs, particularly competence and autonomy, plays a central role in shaping learners' motivation and engagement. In educational contexts, competence refers to learners' perceived effectiveness in performing tasks, whereas autonomy reflects the extent to which learners experience their actions as self-directed and volitional. Although SDT emphasizes the joint importance of these needs, their relative influence may vary across learning environments.

Vocational education represents a context in which competence may play a particularly prominent role. Learning tasks are often highly structured, guided by clear performance criteria, and oriented toward skill mastery. In such settings, learners may rely more on feedback and structured guidance to develop competence, while autonomy may be less salient or operate in a more context-dependent manner.

AI-supported teaching environments provide specific affordances that are likely to influence these psychological processes. Features such as real-time feedback, automated assessment, and performance tracking can enhance learners' perception of competence by making progress visible and reducing uncertainty. At the same time, the impact of AI on perceived autonomy may be mixed, as algorithm-driven guidance can both support and constrain learners' sense of choice.

Against this background, the present study develops a competence-oriented conceptual framework to examine how AI-supported teaching is associated with student engagement in vocational education. Specifically, the study examines whether AI-supported teaching is associated with student engagement and whether this relationship may be indirectly related to perceived competence and perceived autonomy. By doing so, the study provides a competence-oriented interpretation of the potential psychological processes associated with AI-supported teaching in vocational education and offers practical implications for instructional design and educational practice.

## 2 Literature review and hypothesis development

### 2.1 AI-supported teaching in vocational education

AI-supported teaching refers to instructional approaches that integrate artificial intelligence technologies—such as adaptive learning systems, automated feedback tools, and learning analytics—into teaching and learning processes (Holmes et al., 2022; Luan et al., 2020). In recent years, AI applications have been increasingly adopted in higher education and vocational training, with evidence suggesting positive effects on learning efficiency, skill acquisition, and learner satisfaction (Chen et al., 2023; Zawacki-Richter et al., 2019).

Vocational education differs from general academic education in its strong emphasis on practical skills, competency standards, and structured learning progression (Billett, 2011). Learning tasks are typically performance-oriented and closely aligned with real-world professional requirements. These characteristics make vocational education particularly suitable for AI-supported teaching, as AI systems can provide procedural guidance, real-time feedback, and continuous performance monitoring. Such features allow learners to practice skills repeatedly, receive immediate corrective input, and track their progress toward mastery.

However, while the effectiveness of AI-supported teaching has been widely documented, less attention has been paid to the psychological processes through which it influences student engagement. Understanding these processes is essential for explaining not only whether AI-supported teaching works, but also how and why it affects learning behavior.

### 2.2 Student engagement in technology-enhanced learning

Student engagement is commonly conceptualized as a multidimensional construct comprising behavioral, cognitive, and affective components (Fredricks et al., 2004; Wang and Degol, 2019). Behavioral engagement refers to participation and effort, cognitive engagement involves investment in learning and use of strategies, and affective engagement reflects interest and emotional responses to learning activities.

In vocational education, engagement plays a crucial role in both theoretical learning and practical skill development. Engaged learners are more likely to persist in complex tasks, apply effort in skill practice, and achieve higher performance outcomes. Moreover, engagement is not merely an outcome of instruction but also a key mechanism through which instructional innovations exert their effects.

In technology-enhanced learning environments, engagement may be influenced by system design features such as interactivity, feedback, and personalization. AI-supported teaching, in particular, has the potential to enhance engagement by providing timely feedback, adaptive support, and continuous monitoring of learning activities.

### 2.3 Competence and autonomy from the perspective of self-determination theory

Self-Determination Theory (SDT) posits that human motivation and engagement are shaped by the satisfaction of basic psychological needs, particularly competence and autonomy (Ryan and Deci, 2000, 2020). Competence refers to the need to feel effective and capable in one's interactions with the environment, whereas autonomy refers to the need to experience one's actions as self-directed and volitional.

In educational contexts, competence-supportive environments—characterized by clear expectations, structured guidance, and constructive feedback—have been shown to enhance engagement and learning outcomes (Hattie and Timperley, 2007; Shute, 2008). Autonomy-supportive environments, which provide meaningful choices and encourage self-directed learning, also contribute to motivation and engagement, although their effects may vary across contexts (Reeve, 2006; Vansteenkiste et al., 2020).

In vocational education, the relative importance of these needs may differ from that in more open-ended academic settings. Because vocational training is often structured and performance-driven, learners may place greater emphasis on developing competence through guided practice and feedback. In such contexts, autonomy may play a secondary or context-dependent role.

### 2.4 Hypothesis development and conceptual model

AI-supported teaching environments possess features that directly enhance learners' perceived competence. Real-time feedback reduces uncertainty and provides immediate performance information (Hattie and Timperley, 2007; Shute, 2008), while performance tracking makes learning progress visible. In addition, adaptive difficulty ensures that tasks remain challenging yet achievable, thereby supporting mastery experiences. Through these mechanisms, AI-supported teaching may be associated with higher levels of perceived competence, which may in turn relate to student engagement.

The effect of AI-supported teaching on perceived autonomy, however, is less straightforward. While AI systems can offer flexible learning pathways and individualized recommendations, they may also constrain learners' sense of volition by guiding behavior through algorithmic structures. In structured vocational contexts, such constraints may be less salient, as learners typically expect clear guidance and externally defined performance standards. As a result, the influence of autonomy on engagement may be weaker or contingent on contextual factors.

Based on the above discussion, the following hypotheses are proposed:

H1: AI-supported teaching has a significant positive effect on student engagement.

H2: Perceived competence mediates the relationship between AI-supported teaching and student engagement.

H3: Perceived autonomy mediates the relationship between AI-supported teaching and student engagement.

**Figure 1:**
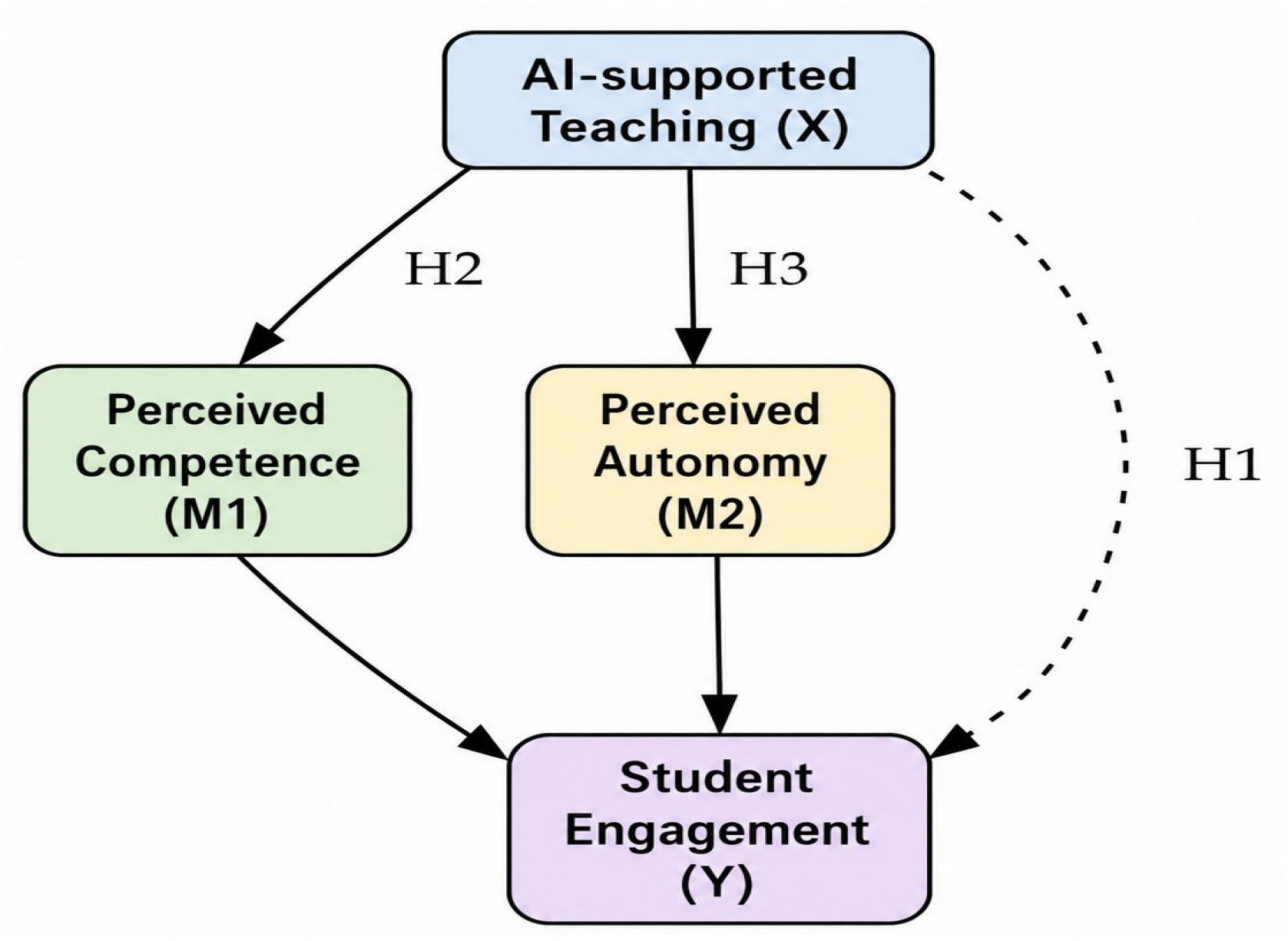
Proposed conceptual framework.

Taken together, these hypotheses reflect a competence-oriented conceptual model in which AI-supported teaching may be associated with student engagement partly through competence-related processes, while the role of autonomy may vary across instructional contexts. Figure 1 summarizes the proposed conceptual framework, in which AI-supported teaching is hypothesized to be associated with student engagement both directly and indirectly through perceived competence and perceived autonomy.

## 3 Methods

### 3.1 Research design and participants

#### 3.1.1 Research design

This study employed a quasi-experimental pretest–posttest control group design over a ten-week intervention period. Because individual random assignment was not feasible in authentic classroom settings, intact classes were used as the unit of assignment. Four existing classes were allocated to either the AI-supported teaching condition or the conventional instruction condition using stratification based on institutional context and baseline academic performance to enhance group comparability.

To enhance internal validity, several controls were implemented. Both the experimental and control groups were taught by the same instructor, followed identical syllabi, and were assessed using the same evaluation criteria. Prior to the intervention, the instructor received structured training to ensure consistency in instructional delivery, feedback practices, and classroom interaction patterns. A standardized teaching script was used to minimize instructor-related variability.

Baseline equivalence between groups was examined using independent-samples *t*-tests across all pre-test variables, including engagement indicators and mediating variables. No statistically significant differences were found (all *p* > 0.05), indicating comparability prior to the intervention.

Given the nested structure of the data (students within classes), clustering effects were additionally examined using supplementary multilevel robustness analyses.

#### 3.1.2 Participants

Participants were 148 second-year vocational college students majoring in preschool education, recruited from three institutions in Sichuan Province, China. The sample reflects typical vocational education contexts and was predominantly female (90.5%), with an age range of 19–22 years (M = 20.3, SD = 0.8).

**Figure 2:**
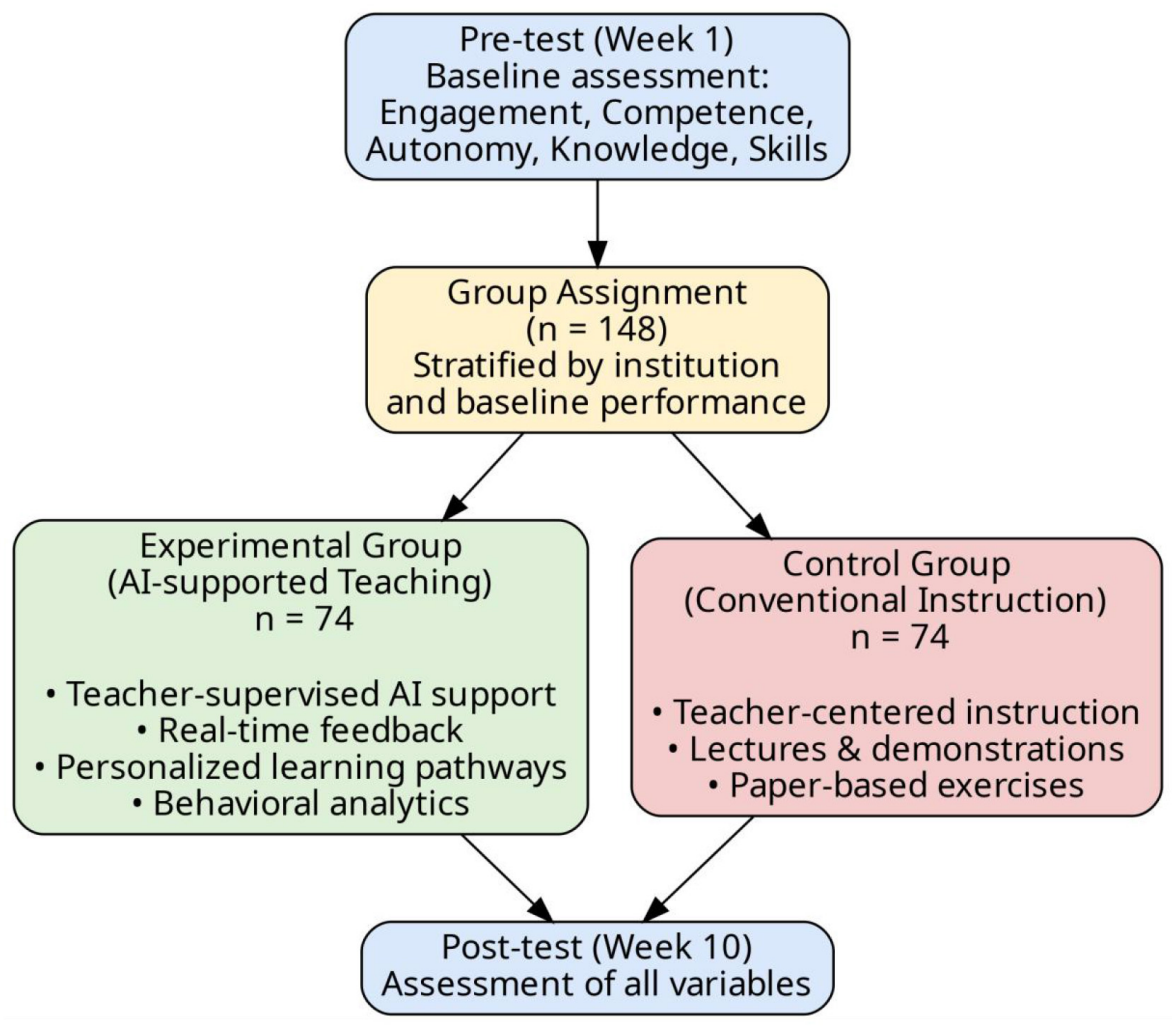
Research design of the quasi-experimental study.

Four intact classes were included in the study, with 74 students in the AI-supported teaching group and 74 students in the conventional instruction group. Figure 2 illustrates the overall research design, including the pre-test, intervention, and post-test phases.

### 3.2 Intervention and instructional conditions

#### 3.2.1 Experimental group: AI-supported teaching

The experimental group participated in an AI-supported instructional program integrated into regular vocational education coursework. The intervention combined teacher-guided instruction with multiple generative AI and intelligent learning tools, including DeepSeek, Kimi, Doubao, and Tongyi Qianwen. These tools were used to support personalized learning, real-time feedback, guided practice, and learning analytics.

Rather than replacing instructors, the AI-supported approach functioned as a teacher-supervised instructional support system. Instructors retained primary responsibility for lesson organization, classroom discussion, instructional explanation, and evaluation of student performance. AI tools were used to supplement teaching activities by providing adaptive learning support and individualized feedback.

The intervention involved four major instructional functions:

1. Real-time feedback and automated assessment Students received immediate task-specific feedback during practice activities. AI-supported systems provided automated scoring suggestions, error identification, and performance analytics. For example, when students completed vocational learning tasks, the system generated instant corrective feedback and highlighted areas requiring improvement.
2. Personalized learning pathways and adaptive recommendations The AI-supported system generated individualized learning recommendations based primarily on students' learning behaviors, including task completion patterns, learning duration, resource access frequency, and prior performance records. Students demonstrating difficulties in specific tasks received additional practice materials, targeted exercises, and supplementary learning resources.
3. Conversational and language-based learning support Students could interact with AI tools through question-answering and dialogic learning activities. AI-supported language generation functions were used to provide explanatory guidance, summarize learning content, and scaffold problem-solving processes.
4. Behavioral learning analytics The platform continuously recorded learning behavior data, including completion rates, time-on-task, frequency of resource access, and interaction patterns. These behavioral indicators were used to monitor engagement and provide adaptive instructional support.

In classroom implementation, students completed a range of vocationally oriented learning tasks, including instructional scenario analysis, lesson-plan design, classroom communication simulations, reflective practice activities, and guided problem-solving exercises related to preschool education contexts. AI-supported tools were used to provide adaptive scaffolding during these activities.

Adaptive recommendations were generated primarily based on students' task completion accuracy, response latency, frequency of repeated errors, resource access patterns, and learning progress indicators. For example, when students repeatedly demonstrated difficulties in specific instructional tasks or showed prolonged response times, the system automatically recommended supplementary practice exercises, review materials, and targeted learning resources aligned with the identified learning needs.

AI-generated feedback typically included corrective suggestions, progress summaries, and task-specific recommendations. Example feedback included statements such as: “You may need additional practice in instructional communication strategies,” or “Your lesson structure is generally appropriate, but classroom interaction techniques could be strengthened.” These feedback messages were intended to support reflective learning and competence development rather than replace teacher evaluation.

To ensure instructional appropriateness and consistency, instructors regularly reviewed AI-generated recommendations and feedback outputs during weekly instructional monitoring meetings. AI-supported outputs that were judged to be unclear, overly generalized, or pedagogically inappropriate were further interpreted and adjusted by instructors before being incorporated into classroom guidance. This teacher-supervised process was implemented to maintain consistency in instructional quality and pedagogical alignment across the intervention period.

The intervention lasted 10 weeks. Students in the experimental group were required to engage with AI-supported learning activities for at least 3 h per week outside regular classroom instruction.

To ensure implementation fidelity, the same instructor taught both experimental and control groups using identical syllabi, learning objectives, and assessment standards. Instructors followed a standardized instructional protocol throughout the intervention period. Weekly monitoring meetings were conducted to ensure consistency in instructional pacing, classroom management, and task requirements.

In classroom practice, instructors supervised students' use of AI tools, facilitated classroom discussion, interpreted AI-generated feedback, and guided reflective learning activities. This design ensured that AI-supported teaching functioned as a pedagogically integrated instructional support system rather than an autonomous replacement for teachers.

#### 3.2.2 Control group: conventional instruction

The control group received conventional teacher-centered instruction consisting of lectures, demonstrations, guided practice, and paper-based exercises. Students completed the same learning tasks, followed the same instructional schedule, and were evaluated using the same assessment criteria as the experimental group.

Unlike the experimental group, the control group did not receive AI-supported feedback, adaptive recommendations, behavioral learning analytics, or personalized learning pathways. Out-of-class learning primarily relied on textbook review, teacher-provided materials, and independent practice activities.

To minimize instructor-related confounding effects, both groups were taught by the same instructor, and all instructional units were implemented within the same 10-week period.

### 3.3 Measures and data sources

A multimodal data approach was adopted to capture both learning outcomes and engagement processes. Table 1 presents an overview of the measurement and reliability information for the key study variables.

**Table 1:** Measurement overview of key variables.

Variable	Type	Measurement	Reliability
Student engagement	Composite	Multi-source indicators (behavioral, cognitive, and affective engagement)	*α =* 0.83
Theoretical knowledge	Test score	Standardized 100-point examination	*α =* 0.86
Practical skills	Performance assessment	Structured rubric with independent ratings	κ = 0.82
Perceived competence	Self-report	5-item Likert scale (adapted from Perceived Competence Scale)	*α =* 0.83
Perceived autonomy	Self-report	5-item Likert scale (adapted from Learning Climate Questionnaire)	*α =* 0.81

#### 3.3.1 Student engagement (dependent variable)

Student engagement was conceptualized as a multidimensional construct comprising behavioral, cognitive, and affective components (Fredricks et al., 2004; Wang and Degol, 2019). This conceptualization reflects the view that engagement represents a multidimensional educational process involving behavioral participation, cognitive investment, and affective involvement in learning activities.

Behavioral engagement was measured using instructor-recorded participation frequency and attendance-related indicators. To ensure objectivity, behavioral data were cross-checked with classroom records and, where applicable, platform usage logs. Inter-rater reliability for behavioral indicators was high (κ = 0.91).

Cognitive engagement was assessed through students' self-reported learning effort, persistence, and use of strategies, supplemented by performance-based indicators such as task completion accuracy and depth of processing. These measures were designed to capture students' investment in understanding and mastering learning content.

Affective engagement was measured using a five-item scale assessing learning interest, enjoyment, and perceived relevance of the learning activities. The scale demonstrated satisfactory internal consistency (Cronbach's α = 0.86).

To construct an overall engagement index, all indicators were first standardized (z-scores) to account for differences in measurement scales across data sources. The standardized indicators were then aggregated to form a composite engagement score. Equal weighting was adopted across the three dimensions because the study aimed to operationalize engagement as a broad educational process integrating behavioral participation, cognitive investment, and affective involvement, rather than emphasizing any single dimension in isolation.

The composite engagement index demonstrated acceptable internal consistency (α = 0.83), suggesting that the multidimensional indicators were sufficiently coherent for use as a pragmatic engagement index within the present study context. This approach allowed the study to integrate multiple data sources and provide a comprehensive measure of engagement in AI-supported learning environments. However, the engagement measure should be interpreted as a multidimensional educational indicator rather than a fully validated latent psychometric construct. Future research may employ confirmatory factor analysis and more comprehensive psychometric validation procedures to further examine the dimensional structure of engagement in AI-supported vocational learning contexts.

#### 3.3.2 Learning outcomes (supplementary variables)

- Theoretical knowledge was assessed using a standardized 100-point examination (Cronbach's α = 0.86).
- Practical skills were evaluated using a structured performance rubric, with independent ratings by two evaluators (κ = 0.82).

#### 3.3.3 Mediating variables

- Perceived competence (5 items, adapted from Perceived Competence Scale; α = 0.83). Example item: “I feel confident in my ability to perform the learning tasks well.”
- Perceived autonomy (5 items, adapted from Learning Climate Questionnaire; α = 0.81). Example item: “I feel that I have a choice in how I complete my learning tasks.”

Both variables were measured using a five-point Likert scale (1 = strongly disagree, 5 = strongly agree).

The present study primarily adopted a pragmatic measurement approach suitable for applied educational research in authentic classroom settings. Internal consistency analyses indicated acceptable reliability for the multi-item measures, with Cronbach's α values ranging from 0.81 to 0.83. In addition, the observed item patterns were generally consistent with the theoretically expected dimensions of perceived competence and perceived autonomy derived from Self-Determination Theory.

Because the engagement construct integrated behavioral, cognitive, and affective indicators from multiple data sources, the present study operationalized engagement as a multidimensional educational index rather than a fully validated latent psychometric construct. Therefore, although the current measures demonstrated acceptable internal consistency and conceptual coherence, future research employing larger samples and confirmatory psychometric procedures is needed to further examine convergent validity, discriminant validity, and measurement structure in AI-supported vocational learning contexts.

#### 3.3.4 Behavioral data (manipulation check)

Behavioral data were collected automatically from the AI platform, including task completion rates, learning duration, and resource access frequency. These data confirmed active platform use (mean usage = 3.4 h/week, SD = 0.7).

### 3.4 Procedure

Data were collected at three time points:

- Pre-test (Week 1): baseline measures of engagement and mediators
- Mid-test (Week 5): interim assessment of cognitive outcomes (not used in main analysis)
- Post-test (Week 10): full assessment of engagement, outcomes, and mediators

Behavioral data were recorded continuously throughout the intervention period. All participants completed the required measures, resulting in a complete dataset.

### 3.5 Data analysis

Data analysis was conducted using SPSS 26.0. The analytical procedure included:

1. Descriptive statistics and reliability analysis
2. Repeated-measures ANOVA to examine intervention effects
3. Hierarchical linear modeling (HLM) to account for clustering effects
4. Mediation analysis using PROCESS (Model 4) with 5,000 bootstrap samples

Indirect effects were considered statistically significant when the 95% confidence interval did not include zero. The direct and total effects were also reported.

To address potential common method bias, both procedural and statistical remedies were applied. Data were collected from multiple sources and at different time points, reducing single-source bias. Harman's single-factor test indicated that no single factor accounted for the majority of variance (< 30%).

### 3.6 Ethical considerations

This study was conducted in accordance with ethical standards for educational research. All participants provided informed consent prior to participation. Participation was voluntary, and participants were informed of their right to withdraw at any time without penalty. All data were anonymized prior to analysis, and no identifying information was collected. The study involved routine instructional practices and complied with institutional ethical guidelines for educational research.

## 4 Results

### 4.1 Preliminary analyses

Descriptive statistics and correlations among the study variables are presented in Table 2. AI-supported teaching (coded 1 = experimental, 0 = control) was positively associated with student engagement (*r* = 0.48, *p* < 0.001) and perceived competence (*r* = 0.53, *p* < 0.001). Perceived autonomy demonstrated a weaker and non-significant correlation with AI-supported teaching (*r* = 0.12, *p* = 0.14).

**Table 2:** Descriptive statistics and correlations (post-test).

Variable	M	SD	1	2	3	4	5
1. AI teaching	0.50	0.50	—				
2. Engagement	3.68	0.72	0.48^***^	—			
3. Competence	3.71	0.75	0.53^***^	0.82^***^	—		
4. Autonomy	3.52	0.73	0.12	0.25^*^	0.27^**^	—	
5. Skills	75.18	9.15	0.51^***^	0.64^***^	0.66^***^	0.18	—

Reliability coefficients for multi-item variables ranged from α = 0.81 to α = 0.86.

^*^p < 0.05, ^**^p < 0.01, ^***^p < 0.001.

Perceived competence was strongly associated with student engagement (*r* = 0.82, *p* < 0.001), whereas perceived autonomy demonstrated a comparatively weaker association (*r* = 0.25, *p* < 0.05). These findings suggest that competence-related processes may be more strongly associated with engagement outcomes within the present instructional context.

### 4.2 Effects of AI-supported teaching on student engagement (H1)

Repeated-measures ANOVA indicated a significant Time × Group interaction effect on student engagement, F_(1, 146)_ = 24.83, *p* < 0.001, partial η^2^ =0.15. Students in the AI-supported teaching group demonstrated greater improvement in engagement across the intervention period than students in the conventional instruction group.

Post-test comparisons further showed that engagement levels were higher in the experimental group (M = 3.68, SD = 0.72) than in the control group, t_(146)_ = 5.12, *p* < 0.001, Cohen's d = 0.84, indicating a relatively large difference between groups.

Taken together, these findings suggest that participation in the AI-supported instructional environment was associated with higher levels of student engagement in the present vocational education context.

### 4.3 Mediation analysis (H2 and H3)

**Figure 3:**
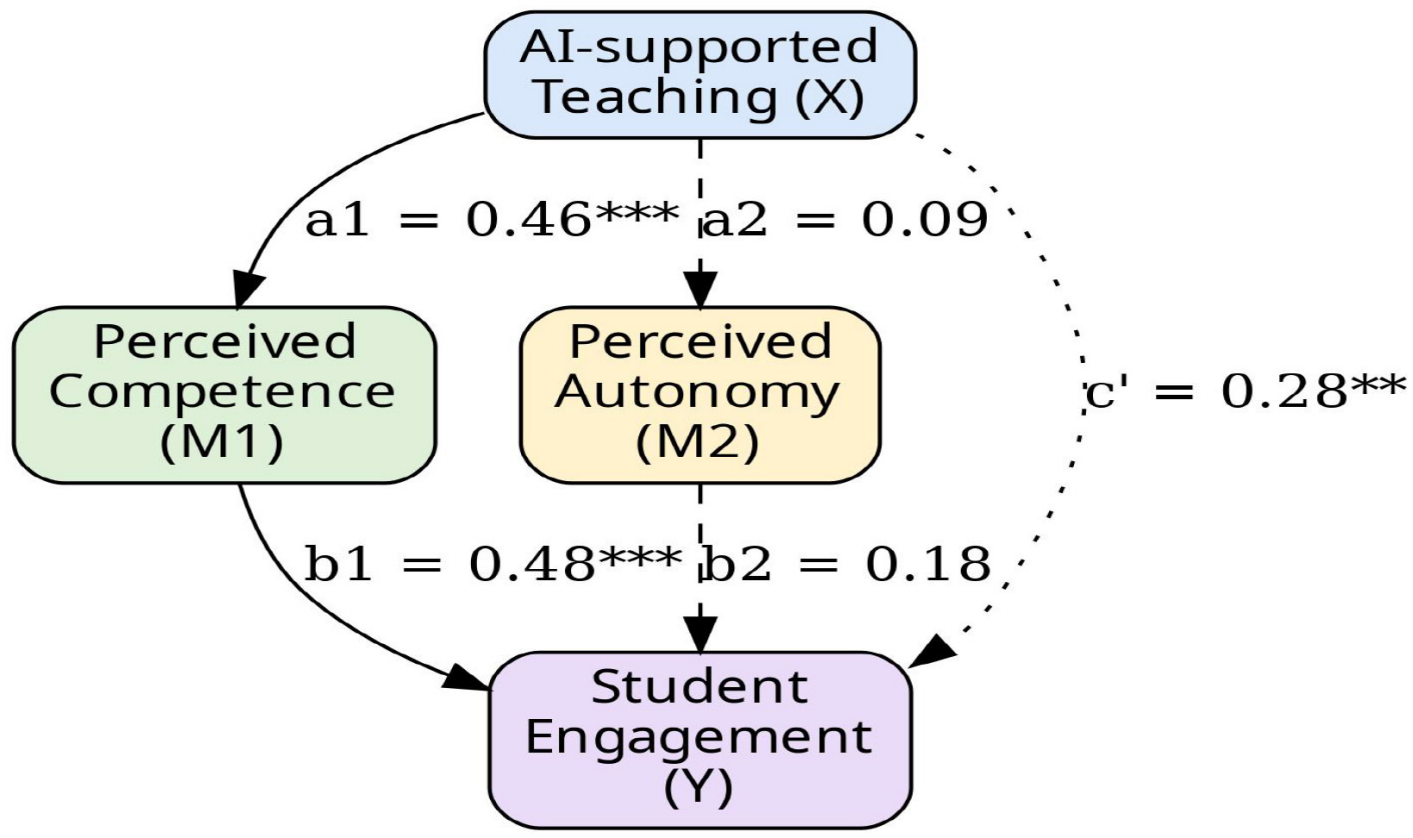
Supplementary mediation analysis results with observed-variable coefficients.

Mediation analysis was conducted using PROCESS Model 4 with 5,000 bootstrap resamples while controlling for pre-test engagement and pre-test mediator variables. The results are presented in Table 3 and Figure 3.

**Table 3:** Mediation analysis results.

Path	β	SE	95% CI	p	Result
AI-supported teaching → perceived competence	0.53	0.07	(0.39, 0.67)	< 0.001	Supported
Perceived competence → student engagement	0.42	0.06	(0.30, 0.54)	< 0.001	Supported
Indirect effect via competence	0.22	0.05	(0.13, 0.31)	< 0.001	Supported
AI-supported teaching → perceived autonomy	0.12	0.08	(−0.03, 0.27)	0.118	Not supported
Perceived autonomy → student engagement	0.15	0.09	(−0.02, 0.32)	0.084	Not supported
Indirect effect via autonomy	0.02	0.02	(−0.02, 0.06)	0.267	Not supported
Direct effect	0.28	0.08	(0.12, 0.44)	< 0.01	—
Total effect	0.52	0.09	(0.35, 0.69)	< 0.001	—

Mediation analysis was conducted using PROCESS Model 4 with 5,000 bootstrap resamples. Pre-test engagement and pre-test mediator variables were included as covariates. β values represent standardized coefficients.

Perceived competence demonstrated a statistically significant indirect association between AI-supported teaching and student engagement [β = 0.22, 95% CI (0.13, 0.31)], providing support for H2. These findings suggest that competence-related experiences may represent one potential mechanism associated with engagement differences observed across instructional conditions.

In contrast, perceived autonomy did not demonstrate a statistically significant indirect effect, as the confidence interval included zero [β = 0.02, 95% CI (−0.02, 0.06)]. Therefore, H3 was not supported within the present sample.

After accounting for both mediators, the direct association between AI-supported teaching and engagement remained statistically significant (β = 0.28, *p* < 0.01), suggesting partial mediation.

Overall, the mediation analyses indicate that perceived competence was more strongly associated with engagement outcomes than perceived autonomy within the present instructional context.

### 4.4 Robustness checks

Several supplementary analyses were conducted to examine the robustness of the findings.

First, a hierarchical linear model (HLM) was estimated to account for the nested structure of students within classes. Although the number of clusters was relatively limited (four classes), the HLM analysis was conducted as a supplementary robustness procedure. The intraclass correlation coefficient (ICC) for post-test engagement was 0.04, indicating relatively small between-class variance. After accounting for clustering effects, the association between AI-supported teaching and engagement remained statistically significant (γ = 0.48, SE = 0.09, *p* < 0.001).

Second, an alternative engagement index using only behavioral indicators produced substantively similar patterns. The indirect association through perceived competence remained statistically significant [β = 0.19, 95% CI (0.10, 0.29)], whereas the autonomy pathway remained non-significant.

Third, sensitivity analyses excluding one class at a time yielded generally consistent results, suggesting that the overall findings were not solely dependent on any single subgroup.

Taken together, these supplementary analyses provide additional support for the overall consistency of the observed patterns, although the findings should still be interpreted within the methodological limitations of the present study.

### 4.5 Supplementary learning outcomes

Although the primary focus of this study is student engagement, additional analyses were conducted to examine learning outcomes.

The results indicated that students in the AI-supported teaching group also demonstrated higher scores in both theoretical knowledge and practical skills than students in the conventional instruction group. Specifically, the experimental group demonstrated higher theoretical knowledge scores than the control group [*M* = 81.45, *SD* = 8.30 vs. *M* = 75.12, *SD* = 8.90; *t*_(146)_ = 4.58, *p* < 0.001, *d* = 0.75], as well as stronger performance in practical skills [*M* = 80.30, *SD* = 7.80 vs. *M* = 70.06, *SD* = 8.40; *t*_(146)_ = 6.28, *p* < 0.001, *d* = 1.04].

These outcomes are reported as supplementary, as they are not central to the hypothesized competence-mediated mechanism. Notably, the largest effect size was observed in practical skills, which may be related to the performance-oriented characteristics of vocational education and the structured features of AI-supported instructional activities.

### 4.6 Exploratory analyses by engagement dimension

Exploratory analyses were conducted to further examine potential differences across engagement dimensions.

AI-supported teaching demonstrated the largest association with behavioral engagement (d = 0.96), followed by cognitive engagement (d = 0.78) and affective engagement (d = 0.71). Perceived competence demonstrated statistically significant indirect associations across all three dimensions, with the strongest indirect pattern observed for behavioral engagement.

These exploratory findings further suggest that competence-related experiences may be particularly relevant for understanding behavioral participation in AI-supported vocational learning environments.

## 5 Discussion

### 5.1 Principal findings

The present study found that AI-supported teaching was associated with higher levels of student engagement in vocational education, with students in the experimental group demonstrating greater post-test improvement than those in the conventional instruction group. The findings further suggest that perceived competence may represent an important psychological pathway linking AI-supported teaching and engagement.

Although the mediation analysis indicated that perceived competence was significantly associated with engagement outcomes, these findings should be interpreted cautiously given the quasi-experimental nature of the study. Rather than establishing definitive causal mechanisms, the results provide preliminary evidence that competence-related processes may help explain how AI-supported teaching relates to student engagement in structured vocational learning environments.

In contrast, perceived autonomy did not demonstrate a statistically significant mediating effect in the present study. This pattern suggests that motivational processes in AI-supported vocational learning contexts may not operate uniformly across psychological dimensions.

Taken together, the findings support a competence-oriented interpretation of AI-supported learning in vocational education while also highlighting the contextual nature of motivational processes.

### 5.2 Theoretical contributions

This study contributes to the literature in several ways.

First, the findings extend discussions surrounding Self-Determination Theory in technology-enhanced learning environments by suggesting that the relative salience of competence and autonomy may vary across instructional contexts. In structured and performance-oriented vocational settings, competence-related experiences may be particularly relevant for explaining variations in student engagement.

Second, the study contributes to the growing literature on AI-supported education by examining potential psychological processes associated with AI-enhanced instructional environments. Rather than focusing exclusively on learning outcomes, the present study explored how instructional features such as real-time feedback, adaptive recommendations, and performance monitoring may relate to students' perceptions of competence and subsequent engagement.

Third, the study adopted a multimodal measurement approach integrating self-report indicators, behavioral records, and performance-based evidence. This approach helps reduce reliance on single-source self-report measures and provides a broader representation of student engagement in AI-supported vocational learning contexts.

However, the findings should be interpreted within the methodological boundaries of the present study design, and future research employing more rigorous longitudinal or experimental approaches is needed to further validate the proposed mechanisms.

### 5.3 Interpreting the non-significant role of autonomy

The non-significant mediating role of perceived autonomy should be interpreted cautiously. One possible explanation relates to the structured characteristics of vocational education contexts. In vocational learning environments, instructional activities are often guided by predefined performance standards, procedural requirements, and clearly specified learning objectives. Under such conditions, students may place greater emphasis on receiving guidance, feedback, and performance support than on experiencing extensive instructional choice.

A second possible explanation concerns the design characteristics of the AI-supported instructional environment used in the present study. Although the system provided adaptive recommendations and personalized feedback, it offered relatively limited opportunities for open-ended decision-making or learner-directed instructional pathways. As a result, the intervention may have supported competence-related experiences more strongly than autonomy-related experiences.

Contextual and cultural factors may also have influenced students' perceptions of autonomy. In educational settings where structured guidance and teacher-directed learning are common, students may respond differently to autonomy-supportive instructional features. However, the present study did not directly examine cultural variables, and therefore such interpretations remain speculative.

Importantly, the absence of a statistically significant mediation effect does not imply that autonomy is unimportant in AI-supported learning environments. Rather, the findings suggest that perceived autonomy did not substantially explain the relationship between AI-supported teaching and engagement within the specific context examined in this study.

Future research may further investigate whether autonomy plays a stronger role in less structured learning environments, longer-term interventions, or instructional settings with greater learner control.

### 5.4 Practical implications

The present findings may provide several practical implications for vocational education and AI-supported instructional design.

First, the results suggest that AI-supported teaching may help support student engagement when instructional systems incorporate timely feedback, adaptive recommendations, and opportunities for guided learning participation. In vocational education contexts, where students often require structured learning support, AI-assisted instructional environments may help facilitate more sustained behavioral and cognitive involvement in learning activities.

Second, the findings indicate that competence-related experiences may be particularly relevant in AI-supported vocational learning settings. Instructional designers and educators may therefore benefit from emphasizing feedback mechanisms, scaffolded learning support, and task structures that help students develop confidence in their learning performance and skill mastery.

Third, the non-significant role of perceived autonomy suggests that simply introducing AI technologies may not automatically enhance students' sense of learner control or instructional choice. Educators may need to more intentionally incorporate learner-directed activities, flexible learning pathways, and opportunities for autonomous decision-making when designing AI-supported instructional environments.

Finally, the findings highlight the potential importance of maintaining teacher involvement within AI-supported learning systems. In the present study, instructors continued to supervise learning activities, guide classroom discussion, and interpret AI-generated feedback. This suggests that AI-supported teaching may function more effectively when integrated into teacher-guided pedagogical practices rather than operating as a fully autonomous instructional system.

### 5.5 Limitations and future research

Several limitations should be acknowledged.

First, the study employed a quasi-experimental design using intact classroom groups rather than individual random assignment. Although baseline equivalence tests and standardized instructional procedures were implemented to reduce potential confounding effects, the design cannot fully eliminate the possibility of instructor effects, classroom-level influences, or novelty effects associated with AI-supported learning.

In addition, students in the experimental group were required to participate in AI-supported learning activities for at least 3 h per week outside regular instructional sessions. Although both groups completed identical curricular objectives and were taught using standardized instructional procedures, differences in learning intensity, feedback exposure, or additional practice opportunities may have partially contributed to the observed engagement differences between groups. Therefore, the findings should not be interpreted as reflecting the effects of AI-supported teaching alone.

Second, the number of classroom clusters included in the study was relatively small. Although supplementary hierarchical linear modeling suggested limited clustering effects, the multilevel analyses should be interpreted cautiously.

Third, the intervention lasted 10 weeks and therefore primarily reflects short-term instructional effects. Future longitudinal studies are needed to examine whether competence-related motivational processes remain stable over extended periods of AI-supported learning.

Fourth, although the study adopted a multimodal measurement approach integrating behavioral, cognitive, and affective indicators, the engagement construct was operationalized as a multidimensional composite index rather than a fully validated latent psychometric construct. Future research may employ confirmatory factor analysis and more comprehensive psychometric validation procedures to further examine the dimensional structure of engagement in AI-supported vocational learning environments.

Fifth, the sample was limited to vocational college students from a specific regional context in China, which may limit the generalizability of the findings to other educational systems or disciplinary settings.

Finally, the present study focused primarily on competence and autonomy as explanatory variables. Other potentially relevant mechanisms, such as relatedness, learning anxiety, self-efficacy, or technology acceptance, were not examined and warrant further investigation.

### 5.6 Conclusion

This study suggests that AI-supported teaching may be associated with higher levels of student engagement in vocational education and that perceived competence may serve as an important psychological pathway linking AI-supported instructional environments and engagement outcomes. Within the present quasi-experimental context, perceived autonomy did not demonstrate a statistically significant mediating effect, indicating that different motivational processes may operate differently across instructional settings.

The findings contribute to ongoing discussions regarding the psychological mechanisms associated with AI-supported learning and highlight the potential importance of competence-related experiences in structured vocational education environments. At the same time, the results should be interpreted cautiously given the quasi-experimental design, the relatively small number of classroom clusters, and the context-specific sample.

Future research employing larger samples, longitudinal designs, and more rigorous psychometric validation procedures is needed to further examine the relationships among AI-supported teaching, motivational processes, and student engagement across diverse educational contexts.

## Data Availability

The original contributions presented in the study are included in the article/supplementary material, further inquiries can be directed to the corresponding author.
